# A New Route for Low Pressure and Temperature CWAO: A PtRu/MoS_2__Hyper-Crosslinked Nanocomposite

**DOI:** 10.3390/nano9101477

**Published:** 2019-10-17

**Authors:** Rachele Castaldo, Mariagrazia Iuliano, Mariacristina Cocca, Veronica Ambrogi, Gennaro Gentile, Maria Sarno

**Affiliations:** 1 Institute for Polymers Composites and Biomaterials, National Research Council of Italy, Via Campi Flegrei 34, 80078 Pozzuoli, Italy; rachele.castaldo@jpcb.cnr.it (R.C.); cocca@ictp.cnr.it (M.C.); gennaro.gentile@cnr.it (G.G.); 2 Department of Industrial Engineering and Centre NANO_MATES University of Salerno, Via Giovanni Paolo II, 132-84084 Fisciano (SA), Italy; maiuliano@unisa.it; 3 Department of Chemical, Materials and Production Engineering, University of Naples Federico II, Piazzale Tecchio 80, 80125 Napoli, Italy; ambrogi@unina.it

**Keywords:** hyper-crosslinked resin, nanocatalyst, phenol removal, catalytic wet air oxidation

## Abstract

PtRu/MoS_2_ nanoparticles (NPs) (PtRu alloy partially coated by one-layer MoS_2_ nanosheets) were prepared through a ‘wet chemistry’ approach. The obtained NPs were directly embedded, at 5 parts per hundred resin/rubber (phr) loading, in a poly (divinylbenzene-co-vinyl benzyl chloride) hyper-crosslinked (HCL) resin, synthesized via bulk polymerization of the resin precursors, followed by conventional FeCl_3_ post-crosslinking. The obtained HCL nanocomposites were characterized to evaluate the effect of the NPs. It shows a high degree of crosslinking, a good dispersion of NPs and a surface area up to 1870 ± 20 m^2^/g. The catalytic activity of the HCL nanocomposite on phenol wet air oxidation was tested at low air pressure (P_air_ = 0.3 MPa) and temperature (T = 95 °C), and at different phenol concentrations. At the lower phenol concentration, the nanocomposite gives a total organic carbon (TOC) conversion of 97.1%, with a mineralization degree of 96.8%. At higher phenol concentrations, a phenol removal of 99.9%, after 420 min, was achieved, indicating a quasi-complete depletion of phenol, with a TOC conversion of 86.5%, corresponding to a mineralization degree of 84.2%. Catalyst fouling was evaluated, showing good reusability of the obtained nanocomposite.

## 1. Introduction

The widespread industrialization process—i.e., the exponential growth of industrial plants—led to a markedly increase in air, soil, and water contaminants concentration. Economic growth related to industrial development is indeed deeply connected to environmental pollution, and strict standards imposed by new legislation as well as the continuously increasing environmental awareness require practical solutions.

Water is a common utility in industrial plants, and the risk of contamination is unavoidably high. An enormous number of organic compounds, widely used in industrial plants (petrochemical, pharmaceutical, bleach, dye, etc.), are extremely toxic contaminants. In order to meet requirements for discharging or recycling, wastewaters should be adequately treated to deplete concentrations of hazardous molecules. Among them, phenol and its derivatives represent a class of treacherous and high biological dangerous compounds [1,2,3], that seriously threaten biological life at high concentrations. Indeed, phenol oxidation follows a complex series of reactions [4,5,6,7], through intermediate compounds increasing potential accumulation.

The drawback of the typical phenol reduction processes [8,9] is that they do not allow a phenol conversion into less dangerous compounds, resulting in a global phenol accumulation. WAO (wet air oxidation) is a well-known method for wastewater treatment, in which highly reactive hydroxyl radical species forming in the reaction environment oxidize organic compounds. However, the process, despite its intrinsic design simplicity, is limited by the prohibitive conditions of pressure, i.e., up to 20 MPa, and temperature, i.e., up to 300 °C [10,11,12]. Heterogeneous catalysis is one of the most effective strategies to improve process efficiency removal [13,14,15,16,17,18,19]. This is particularly true considering the major drawbacks of homogenous catalysis and the problems related to the utilization of biological treatment for highly contaminated wastewater. Many species have been extensively tested as heterogeneous catalysts in catalytic wet air oxidation (CWAO) (e.g., noble metals [20,21,22,23,24,25,26,27,28,29,30], metal oxides [31,32,33,34,35,36,37,38], carbon materials [39,40,41,42,43]), in a wide range of conditions, with noble metals showing the highest activity. Among noble metals, platinum and ruthenium have been reported as excellent catalysts towards phenol oxidation. Moreover, a great variety of supports, to disperse and stabilize active phases, and operating at different conditions of temperature (100–160 °C) and pressure of air (1.5–5.6 MPa), have been investigated for phenol CWAO [20,21,26,28,29,35,36,39,44,45]. Remarkable conclusions can be derived: catalyst poisoning (e.g., sulphur-containing compounds, phosphorous) is a crucial factor ensuring activity; reactions, in particular, high reactants concentration, led to catalyst deactivation due to polymer surface covering; selectivity to CO_2_ is influenced by pressure conditions; high temperatures, up to 160 °C, should be reached in order to enhance reaction rates.

Nanotechnology, enabling the perfect control over shape and dimensions of nanocrystals forming in solution, represents a way of innovation for heterogeneous catalysis, with improved diffusions and high surface area exposed (i.e., quasi-homogeneous catalysis). However, the widespread utilization of solid nanoparticles (NPs) in catalysis is limited by aggregation phenomena involving NPs due to the large surface area exhibited in colloidal suspension.

Hyper-crosslinked (HCL) polymers can represent an innovative possible solution to this problem, offering the opportunity to embed nanoparticle directly in a porous, chemically stable, polymeric matrix. HCL resins (also called Davankov resins) are microporous polymers prepared from a wide range of aromatic monomers, by crosslinking of a linear precursor or a light crosslinked gel-type polymer matrix, via a two-step or one-pot synthetic procedure [46,47,48]. These are an exciting class of materials with a high ability to adsorb chemicals [49]. Nevertheless, in the past years, the application of NPs loaded HCL has been limited by complex synthetic procedures [46].

This work concerns the preparation of an innovative nanocatalyst, namely PtRu/MoS_2_ nanoparticles, the development of a new route for dispersion of this nanocatalyst in a high surface area HCL resin and the test of this system for the removal of phenols in water in extremely mild conditions.

In particular, uniform size, opportunely surface-modified PtRu/MoS_2_ nanoparticles, constituted of PtRu alloy NPs partially covered by MoS_2_ nanosheets, were obtained through a scalable and reproducible ‘wet chemistry’ synthetic approach [50,51]. For what concerns the resin, here we propose the synthesis of styrene-based HCL resin via a modified Davankov synthetic strategy, in which a precursor polymer is prepared by bulk polymerization and then hyper-crosslinked by Friedel–Crafts reaction [52]. Through this procedure, microporous nanocomposites were already realized, containing homogeneously dispersed nanomaterials such as carbon nanotubes, graphene nanoplatelets, or graphene oxide [47,52]. Therefore, this procedure was applied to the preparation of microporous nanocomposites containing PtRu/MoS_2_ NPs, allowing them to obtain a high surface area nanocomposite containing homogeneously dispersed nanoparticles. The final aim of this work was to investigate the catalytic activity of PtRu alloy NPs and their MoS_2_ nanosheets protective layer, stabilized and dispersed in an HCL porous resin, for phenol CWAO at low air pressure and 95 °C. It is worth noticing that, although the preparation of the nanocatalyst and nanocomposite will require effort to implement production lines at an industrial level, the reduced amount of catalyst, which is in fact produced in mild conditions and through a scalable process [53,54,55]; the fact that the synthesis of hyper-cross-linked resins is an established low-cost mass production process [48,52,56,57], here proposed to prepare in a one-step the catalytic nanocoposites; and, finally the mild conditions required by the nano-composite for pollutant removal, make this approach promising. Moreover, excellent removal performance was obtained. This is due to a combination of factors: the preservation of the hierarchical structure of the resin, enabling easy access to the pores; the NPs confinement and reagents pre-concentration in the adsorbent resin; PtRu alloy activity; the MoS_2_ highly defective nanosheets protecting the catalyst surface against carbon polymerization and poisoning.

## 2. Materials and Methods

### 2.1. Materials

Platinum (III) acetylacetonate (97%), ammonium tetrathiomolybdate (>99%), ruthenium (III) acetylacetonate (>97%), oleic acid, oleylamine, 1,2-hexdecanediol, 1-Octadecene, hexane, phenol, vinylbenzyl chloride (VBC, >95%, mixture of isomers, ∼70% meta + ∼30% para), *p*-divinylbenzene (DVB, 85%, meta isomer ∼10 wt %), 2,2′-azobis(2-methylpropionitrile) (AIBN, >98%), FeCl_3_ (>97%), phenol (>99.5%), 4-aminoantipyrine (AAP)**,** potassium ferricyanide and sodium bicarbonate. All reagent were purchased by Sigma-Aldrich (Milan, Italy), and used without further purifications.

### 2.2. Synthesis of the Nanoparticles

Platinum (III) acetylacetonate (1.271 mmol), and ruthenium (III) acetylacetonate (0.753 mmol) were used as metal precursors. Ammonium tetrathiomolybdate (1.153 mmol) was used as a molybdenum disulphide precursor. Oleic acid (6 mmol), oleylamine (6 mmol), and 1,2-hexadecandiol (10 mmol) inappropriate amount were used as surfactants and reducing agent, respectively. 20 mL of high boiling point 1-octadecene was used as a reaction solvent. The reagent mixture was heated up to 200 °C for 2 h, and then the temperature was rapidly increased to 285 °C for 1 h. Reagents excesses were removed by centrifugation-redispersion method, alternating ethanol, and hexane washing. Purified nanoparticles of surface-modified hydrophobic PtRu/MoS_2_ were re-dispersed in hexane and dried.

Nanoparticles of Pt/Ru and MoS_2_ nanosheets were also prepared in the same operating conditions and reagents, and in the presence of appropriate precursors. Before use, they were treated at 150 °C for 5 h to remove chains of surfactants.

### 2.3. Synthesis of the Nanocomposite

The hyper-crosslinked poly(divinylbenzene-co-vinyl benzyl chloride) based nanocomposite containing PtRu/MoS_2_ nanoparticles were prepared through a two-step procedure. DVB and VBC (molar ratio 2:98) were mixed with 5 parts per hundred resin/rubber (phr) of PtRu/MoS_2_ nanoparticles. To ensure effective nanofiller dispersion, the mixture was sonicated for 50 min with a 500 W tip sonicator (UP 400S, Hielscher, Teltow, Germany) at 25% power, with a 10 s/50 s ON/OFF cycle. Therefore, 0.5 phr of AIBN was added, and the mixture was kept under stirring at constant temperature (80 °C) under nitrogen for 30 min. Polymerization was completed in an oven for 24 h at 80 °C. For comparison, neat poly(divinylbenzene-co-vinyl benzyl chloride) was prepared similarly. The obtained nanocomposite and polymer precursors were repeatedly washed with methanol, acetone, and diethyl ether, and then dried in a vacuum oven (Gongyi Yuhua Instrument Co., Ltd., Gongyi, China) at 40 °C for 24 h.

For the synthesis of the hyper-crosslinked systems, both precursors were swollen in 1,2-dichloroethane for 2 h, then the systems were cooled to 0 °C using an ice/water bath, FeCl_3_ was added, and stirring was continued for 2 h. After that, the reaction flask was heated to 80 °C and kept at this temperature for 18 h. The obtained hyper-cross-linked resin and nanocomposite were washed with methanol and dried in a vacuum oven at 40 °C.

### 2.4. Catalytic Tests

Catalytic tests in phenol wet air oxidation were conducted in a glass semi-batch apparatus. The reaction system, magnetically stirred and temperature-controlled, was equipped with a heating jacket stage. Gas feeding was monitored and controlled by a gas flow controller. In this set of experiments, pure air (SOL 99.999%) and pure nitrogen (SOL 99.999%) were used. In a typical run, 10 mL of aqueous phenol solution at known concentration were placed into the reactor, following catalyst loading. The temperature was increased, up to the operating condition, under N_2_ gas to avoid uncontrolled degradation phenomena. When the set point was reached, gas feed changed to compressed air, allowing the gas to bubble throughout the liquid batch. The constant gas flow of 4.6 NL/h and stirring were maintained during the experiment. Samples were taken at various time intervals, and phenol concentrations or UV–vis spectra were acquired. Operating conditions were summarized in Table 1. Moreover, before all the catalytic tests, the resins (0.4 g/L), loaded and not with the catalyst, were saturated with phenol in the absence of airflow, at 95 °C, and in a 4000 mg/L solution of phenol, waiting for equilibrium and then taken for the tests performance.

The CO_2_ evolved was monitored with a SIEMENS Utramar 22 analyzer (Siemens, Erlangen, Germany). The CO_2_ of the liquid phase in equilibrium with the gas phase was obtained from specific equilibrium studies at the pressure and temperature of the tests.

Gas-chromatographic analyses were performed in a GC Focus Series (Thermo Scientific) coupled with a single quadrupole ISQ (Thermo Scientific, Waltham, MA, USA) mass spectrometer. The method details are summarized in Table 2. Acylation of the reaction solutions was performed for analysis.

Total organic carbon values were analyzed in a 1020A TOC analyzer, the removal ratio Δ(TOC)% was expressed as in the equation

Δ(TOC)% = (TOC_0_ − TOC_1_)/TOC_0_ × 100 (1)

Mineralization degree was evaluated through the equation

M% = [CO_2_]_tot(t)_/TOC_0_ × 100 (2)

The carbon contents in catalysts after tests were evaluated by temperature-programmed oxidation (TPO) using a 1% O_2_/He mixture (14 mL/min) in the range 30–700 °C temperature range (heating rate: 70 °C/min).

Turn over frequency (TOF) values were evaluated through the equation

TOF = C_PheR_/C_cat_ × t (3)

where C_PheR_ is the concentration of phenol removed; C_cat_ is the concentration of catalyst; and, t is the reaction time.

### 2.5. Characterization Methods

Bright-field transmission electron microscopy (TEM) analysis was performed on the PtRu/MoS_2_ NPs and the nanocomposite precursor using a FEI Tecnai G12 Spirit Twin (LaB6 source, Eindhoven, The Netherlands). at 120 kV acceleration voltage. Before analysis, the PtRu/MoS_2_ nanoparticles were dispersed in toluene by sonication and collected on holey carbon-coated copper grids, and ultrathin sections of the nanocomposite precursor were prepared with a Leica UC7 ultramicrotome (nominal thickness 100 nm) and deposited on TEM copper grids. TEM images were acquired by a FEI Eagle 4k CCD camera (Eindhoven, The Netherlands).

Specific surface area (SSA) and pore size distribution analysis were performed on the hyper-crosslinked resin and nanocomposite by nitrogen adsorption measurements at 77 K, using a Micromeritics ASAP 2020 analyzer (Norcross, GA, USA). Before the analysis, all the samples were degassed at 120 °C under vacuum (*p* < 10^−5^ mbar) and the measurements were performed using high purity gases (>99.999%). Brenauer–Emmett–Teller (BET) SSA was determined by the linear part of the BET equation. Nonlocal density functional theory (NLDFT) was applied to the nitrogen adsorption isotherms to evaluate the pore size distribution of the materials. Phenol concentrations were measured by the colorimetric method by using a Thermo-Scientific UV–vis Evolution Q60 spectrophotometer. Aliquots (800 µL) of the diluted sample were placed in a spectrophotometer cuvette (1 mL) with 100 µL AAP solution (20.8 mM in 0.25 M sodium bicarbonate solution) and 100 µL potassium ferricyanide solution (83.4 mM in 0.25 M sodium bicarbonate solution). Absorbance was measured at 510 nm against a blank. Concentrations were measured using a previously recorded calibration curve (mg/mL of phenol = 0.0195 x Abs + 7 × 10^−5^, R² = 0.9999). Thermogravimetric analysis (TG-DTG) (SDTQ 600 Analyser (TA Instruments, New Castle, DE, USA) was performed in flowing air at a 10 K/min heating rate. FT-IR spectra (Vertex 70 apparatus, Bruker Corporation, Billerica, MA, USA), by applying KBr technique, X-ray diffraction measurements (Bruker D8 X-ray diffractometer using CuKα radiation) were also performed.

## 3. Results

### 3.1. Nanocatalyst Characterization

**Figure 1:**
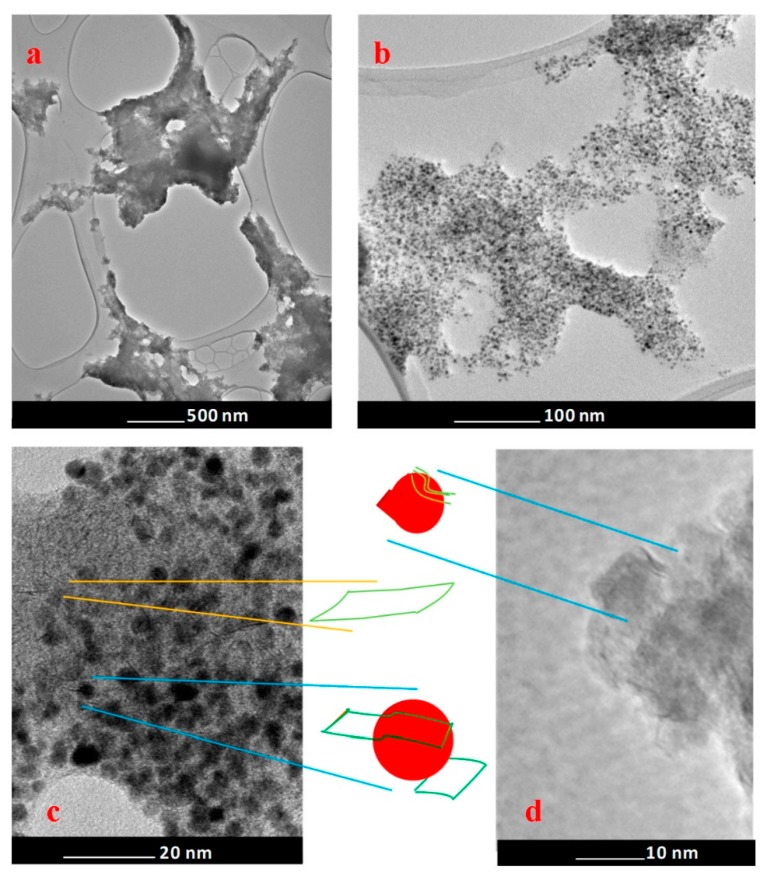
TEM images of the as-prepared PtRu/MoS_2_ nanocatalyst at different magnifications.

Bright-field TEM images at different magnifications of the nanohybrid obtained are shown in Figure 1. The figures allow evaluating the morphology of the as-prepared PtRu/MoS_2_ nanoparticles, constituted of PtRu NPs (mean size 4 nm–standard deviation 0.8 nm) which are partially covered by MoS_2_ nanosheets, mostly 1 layer (Figure 1d) [58].

In the sample MoS_2_ nanosheets alone can also be observed. The Pt/Ru atomic ratio, obtained by energy dispersive TEM based X-ray spectroscopy (EDS), was ~1.94, which is consistent with the precursor’s concentration. The atomic ratio between S/Mo results equal to ~2.1, indicating the presence of low excess sulfur.

**Figure 2:**
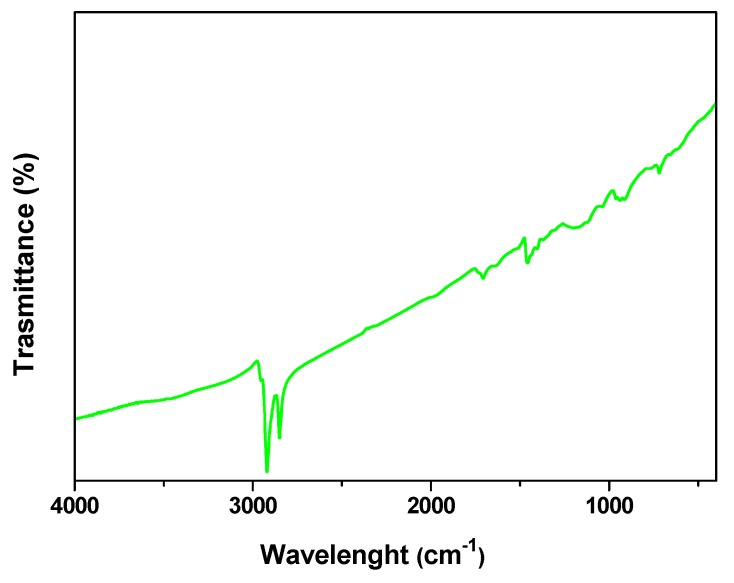
FT-IR spectrum of the so-prepared nanoparticles.

The presence of the organic capping is evidenced by the characteristic FTIR adsorption bands (Figure 2) of the oleic group in the 2850–3000 cm^−1^ region, the ν(C = C) stretch mode at 1647 cm^−1^, and the peak at 1468 cm^−1^ due to the (C–H) bending mode.

In the IR spectrum of the nanohybrid, the absence of free oleylamine is indicated by the lack of –NH_2_ bending mode at 968 cm^−1^, of N–H stretching mode at 3319 cm^−1^ typical of free primary amine and of –NH_2_ scissoring mode at 1560 cm^−1^ (see Figure S1) [51]. The presence of the oleic acid capping is suggested by the weak bands at 1541cm^−1^ and 1649 cm^−1^ [59]. Moreover, the presence of free oleic acid was observed; see the band centered at 1710 cm^−1^, which indicates a lower affinity of this surfactant. On the other hand, the free acid chains can be easily washed away, whereas a contribution during the synthesis to the micelles formation, leading to such small particles, cannot be neglected.

**Figure 3:**
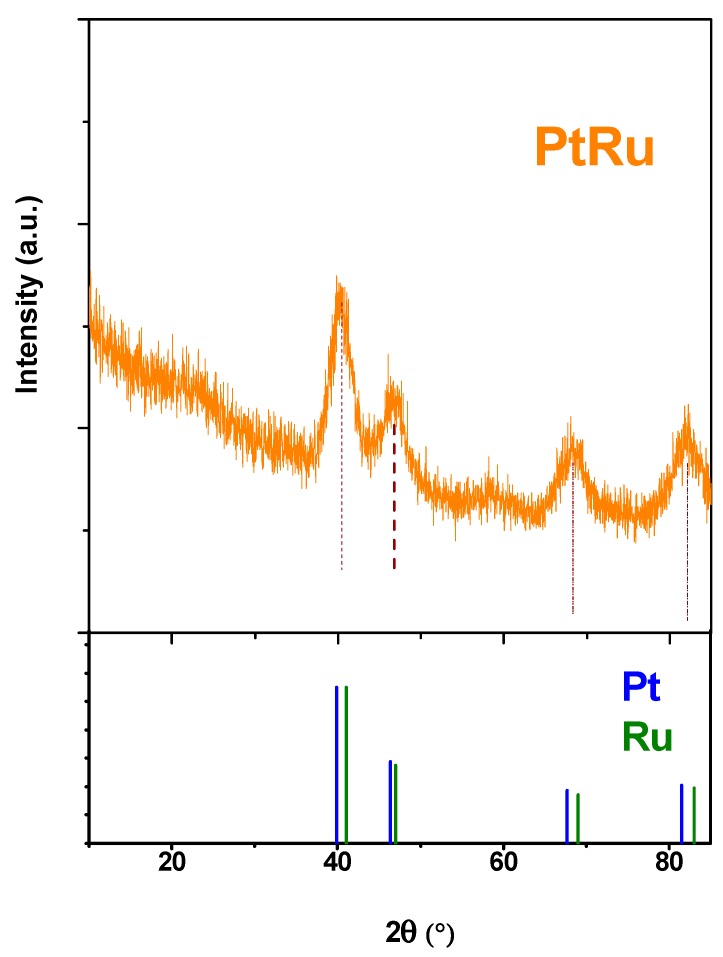
XRD spectrum of PtRu/MoS_2_ nanoparticles.

Figure 3 shows the X-ray diffraction pattern of the nanoparticle synthesis product after a suitable washing. The diffraction peaks of platinum face-centered cubic (fcc) structure, up-shifted due to the incorporation of Ru atoms indicating the formation of an alloy, are clearly visible in Figure 3. The reflection at about 60° can be assigned to the MoS_2_ nanosheets structure [60]. The spectrum of the MoS_2_ is almost completely covered by the metal alloy pattern; this is probably due to the non-crystalline structure of the one-layer MoS_2_ nanosheets typically formed by this process [61].

### 3.2. Resin Characterization

HCL resin and HCL nanocomposite containing PtRu/MoS_2_ NPs were prepared by bulk polymerization of the precursor polymer and nanocomposite, followed by extensive Friedel–Crafts alkylation [52].

**Figure 4:**
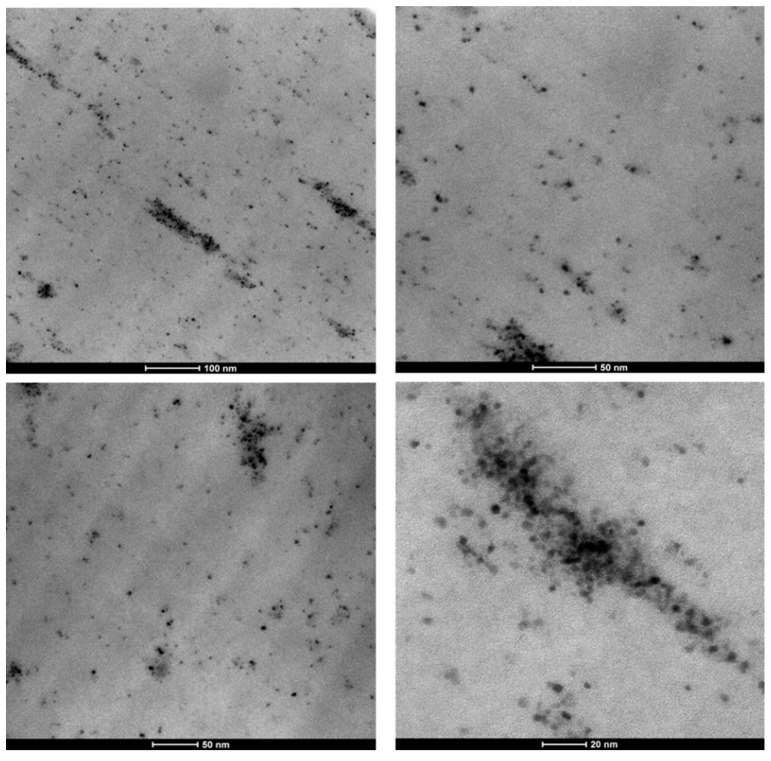
TEM images of dispersed nanoparticles in the polymeric resin.

The distribution of the NPs in the precursor nanocomposite was evaluated by TEM analysis. The HCL nanocomposites, containing PtRu/MoS_2_ NPs, show the good distribution of the nanoparticles, even though minor agglomeration phenomena are present, see Figure 4.

**Figure 5:**
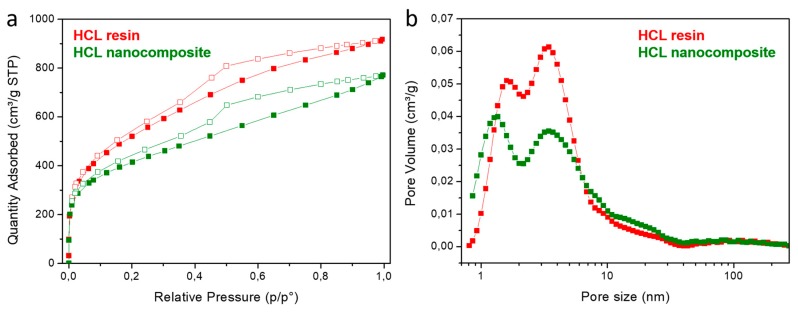
Nitrogen adsorption (filled symbols) and desorption (empty symbols) isotherms at 77 K (**a**) and DFT pore size distribution (**b**) of the HCL resin and HCL nanocomposites.

SSA and porosity distribution of the hyper-cross-linked resin and nanocomposite were also measured using nitrogen volumetric gas adsorption. Both samples show a type II isotherm for nitrogen adsorption at 77 K, with hysteresis during the desorption step, due to the presence of a mesoporous fraction (Figure 5a) [62].

The neat HCL resin and the HCL nanocomposite containing PtRu/MoS_2_ NPs exhibit BET SSA of 1870 ± 20 m^2^/g and 1370 ± 20 m^2^/g, respectively, and the nanocomposite shows a total pore volume about 16% lower than the neat HCL resin. By NLDFT, the pore size distribution of the two samples was investigated (Figure 5b), revealing a similar pore size distribution, characterized by major peaks centered around 1.3–1.6 nm and 3.4 nm. In the HCL nanocomposite, an increase of the micropores/mesopores ratio is observed with respect to the neat resin. This change of the porosity distribution was already observed in other hyper-crosslinked nanocomposites [47,52] and can be explained on the basis of a slightly lower hyper-crosslinking extent obtained in nanocomposite due to the hindrance effect of the NPs. This is similar to the effect reported for different functional systems in which part of the chloromethyl groups of the resins was replaced by non-reactive moieties [63].

**Figure 6:**
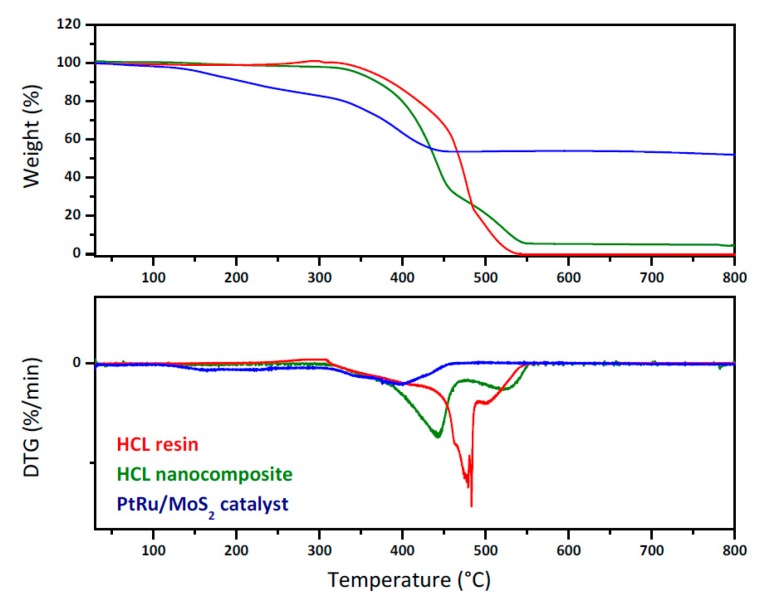
TG-DTGA analysis of PtRu/MoS_2_ nanoparticles, HCL resin, and HCL nanocomposite.

The thermal conversion of PtRu/MoS_2_ in airflow occurred in two main weight loss steps (Figure 6). At 125 °C the oxidation/decomposition of the organic capping of oleylamine and oleic acid chains starts together with a SO_2_ release [58,59] followed by the oxidation of MoS_2_ to MoO_3_ with additional SO_2_ release. The thermogravimetric profiles of neat resin and neat resin loaded with the catalyst are shown in the same figure. The thermogravimetric profiles of the neat and loaded resins are different because of the presence of the catalyst nanoparticles, which contribute to the residue shown by the green profile for loaded resin. In particular, the loaded resin shows slightly lower stability, likely due to the catalytic oxidation behavior of the loaded nanoparticles [64].

### 3.3. Catalyst Performance

**Figure 7:**
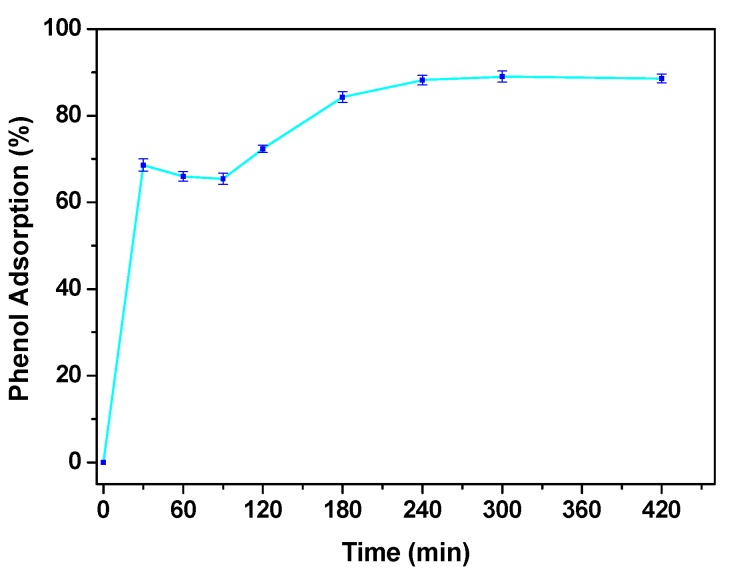
Phenol adsorption performance at C_phenol_ = 4000 mg/L with HCL resin C_Solid_ = 0.4 g/L.

First of all, to analyze the behavior of the neat resin it was tested in the operating conditions: 95 °C, phenol concentration 4000 mg/L, resin concentration 0.4 g/L. The tests evidence the capability of the system to remove more than 60% of the phenol in 30 min, see Figure 7.

**Figure 8:**
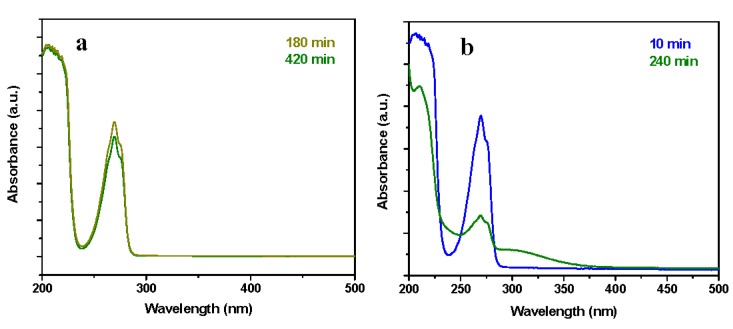
UV-spectra of the reaction media (reaction solutions at specific times) at 95 °C, 4000 mg/L phenol concentration, 0.4 g/L of HCL resin (**a**) and HCL nanocomposite (**b**).

In particular, the removal ability of the resin reached a plateau of ~88% phenol removal after 240 min. To distinguish between adsorption and catalytic behavior, in Figure 8a the UV–vis spectra at 180 min and 420 min of the solution media, showing the adsorption of phenols (i.e., reduction of the intensity of the absorbance peaks) and the absence of chemical reactions, were reported. This result was further analyzed by GC-MS, which shows, for the tests up to 420 min, a single peak at 19.95 min retention time, attributable to phenol.

**Figure 9:**
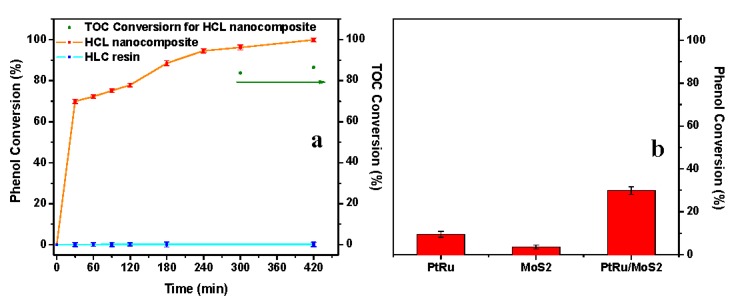
(**a**) Results of CWAO phenol oxidation with HCL resin and HCL nanocomposites. Reaction condition: temperature, 95 °C; pressure, 0.3 MPa; air gas flow, 4.6 NL/h; concentration of solid, C_Solid_ = 0.4 g/L; phenol concentration, 4000 mg/L. (**b**) Results of CWAO phenol oxidation with PtRu, MoS_2_ and PtRu/MoS_2_ after 180 min. Reaction condition: temperature, 95 °C; pressure, 0.3 MPa; air gas flow, 4.6 NL/h; concentration of catalyst, C_cat_ = 0.02 g/L; phenol concentration, 4000 mg/L. Before starting the tests, catalyst loaded and unloaded resins were saturated with phenol in the absence of the airflow at 95 °C (i.e., 4000 mg/L was the starting phenol concentration for the tests collected in this graph).

**Figure 10:**
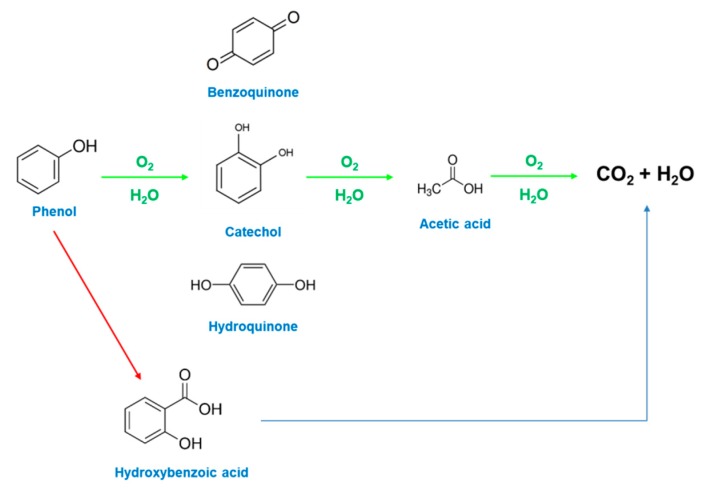
Scheme of phenol wet air oxidation.

Following this experiment, neat and catalytic resins were tested in the same operating conditions but after saturation with phenol, evidencing a significant phenol removal efficiency for the nanocomposites in comparison with an almost total absence of catalytic activity for the unloaded resin, see Figure 9a. Although, after 10 min, in the case of catalyst loaded resins, see the blue profile in Figure 8b, no significant catalytic activity is observed, at 240 min the formation of acetic acid at lower wavelengths and of hydroquinone at higher wavelengths can be seen, see the green spectrum in Figure 8b. In this case, the analysis by GC evidence, e.g., that at 300 min the phenol content was 30.01%; the remaining 69.99% consists of hydroquinone 2.98%, *p*-benzoquinone 0.41%, and acetic acid 96.61%, see the scheme in Figure 10. Because of the maximum phenols content in the solution, at this time, is equal to 5.43%, see Figure 9a, the total phenols content still presents in the solution after 300 min account for a maximum of 217 mg/L, which gives a total carbon content of 727 mg/L, which means a carbon removal of 81.8%. After 300 min, TOC conversion (ΔTOC) evaluation shows a result equal to 83.8%. The slight difference in the carbon removal efficiency obtained with GC/UV–vis and TOC evaluations is probably because of other species—e.g., hydroquinone—contribute, although weakly [65], to the 5.43% phenol residue evaluated through UV–vis. After 420 min a phenol removal of 99.9% was achieved indicating a quasi-complete depletion of phenol. In these conditions, a TOC conversion (ΔTOC) of 86.5%, with a mineralization degree of 84.2%, was measured. At this time the phenol content in the reaction mixture was about 6.1% and the remaining ~ 93.9% consists of acetic acid.

During phenol CWAO, hydroquinone is the primary intermediate, which is quickly oxidized to *p*-benzoquinone. It evolves mainly towards CO_2_, while a lower amount oxidized in short-chain acids to, finally, give acetic acid and CO_2_. We believe that we found for a longer time the presence essentially of acetic acid because it is a very refractory compound in CWAO conditions, even more at the soft conditions of our study, as it requires drastic conditions to be oxidized at some extent. Indeed, acetic acid can be considered a product in CWAO together with CO_2_ [66,67].

In Figure 9b, the results of catalytic tests for the abatement of phenol at a concentration of 4000 mg/L, and after 180 min reaction time, carried out in the presence of the nano-catalysts alone are reported. The role of resin, in the low oxygen atmosphere of the tests, promoting catalyst activity is evident. Although the phenol removal by using PtRu/MoS_2_ alone was of about 29.89% after 180 min and increases of about 20% up to 420 min reaction time, it stays lower than 40%, likely due to a poor oxygen supply. Moreover, PtRu has an even more contained catalytic activity. The TOF values after 180 min reaction time for PtRu/MoS_2_, PtRu/MoS_2_ loaded resin and PtRu are 2.7 x 10^−2^, 7.9 x 10^−2^ and 0.5 × 10^−2^, respectively, highlighting the effect of the catalyst dispersion in the resin. On the other hand, molybdenum disulphide is not a catalyst for phenol oxidation. Indeed, after 180 min in the same conditions as in the experiments of Figure 9b, but at room temperature, it still removes about 2.9% of phenol. On the contrary, the simple adsorption on PtRu particles after 180 min is negligible. The non-obvious role of MoS_2_ is highlighted by the results shown in Figure 9b, too. In particular, cyclic experiments were carried out to evaluate the recyclability of the catalysts on the respect of catalyst fouling [68]. PtRu and PtRu/MoS_2_, after regeneration, were submitted, in the same experimental conditions of Figure 9b, to cycling tests. For the regeneration, the eventually present carbonaceous species were removed in a tubular reactor [69,70] with a flow of 4 *v/v*% of O_2_ in N_2_, 25 mL/min at 280 °C. The samples were further reduced for 30 min under a flow of H_2_ 5 *v/v*% in N_2_, 25 mL/min at 280 °C) before the reuse. The mmolg^−1^ of carbonaceous deposit measured by TPO, with an error of about 10%, was of 12.3 and 1.2 for PtRu and PtRu/MoS_2_, respectively, evidencing the role of MoS_2_ in physically counteracting the carbon deposition. In particular, because of molybdenum disulphide does not completely cover the catalyst; its surface continues to be active. Moreover, the presence of numerous defects in the MoS_2_ nanosheets probably acts as an oxygen reservoir [71] favoring the conversion process and, overall, helping the maintenance of the catalytic activity. The phenol removal %, in a successive cycle at the same operating conditions, was maintained for both regenerated PtRu and PtRu/MoS_2_ at about 99% of the first cycle, after 180 min. On the other hand, without regeneration, the phenol removal was reduced, during the second cycle of use, by about 50% and 3% for PtRu and PtRu/MoS_2_, respectively. In particular, further experiments, performed for a larger number of cycles, show that an activity reduction of about 8% was observed after 10 cycles of use, indicating good stability for the catalyst in the presence of MoS_2_ protective nanosheets.

Further tests were performed to evaluate the catalytic activity during cycles of reuse for the catalyst loaded resins. The catalytic resin was reused, after washing in isobutanol and drying [72], in the same experimental conditions of Figure 9a,b. Maintenance of about 96% and 80% catalytic activities were observed after 300 min of reuse without other regeneration operation, during the 2nd and 10th cycle, respectively. Although the industrial scale will require regeneration cycles, they will be less frequent and laborious than for adsorbent materials, e.g., they could be performed in situ just by increasing temperature and feeding gas. New experiments, e.g., in continuous operation, are now under evaluation.

**Figure 11:**
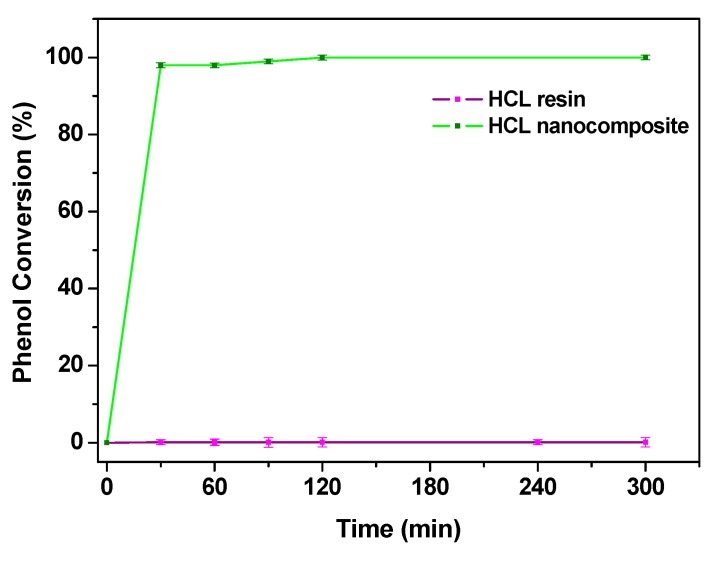
Results of CWAO phenol oxidation with HCL resin and HCL nanocomposite. Reaction condition: temperature, 95 °C; pressure, 0.3 MPa; air gas flow, 4.6 NL/h; concentration of solid, C_Solid_ = 0.4 g/L; phenol concentration, 1000 mg/L. Before starting the tests, catalyst loaded and unloaded resins were saturated with phenol in the absence of the airflow at 95 °C (i.e., 1000 mg/L was the starting phenol concentration for the tests collected in this graph).

**Figure 12:**
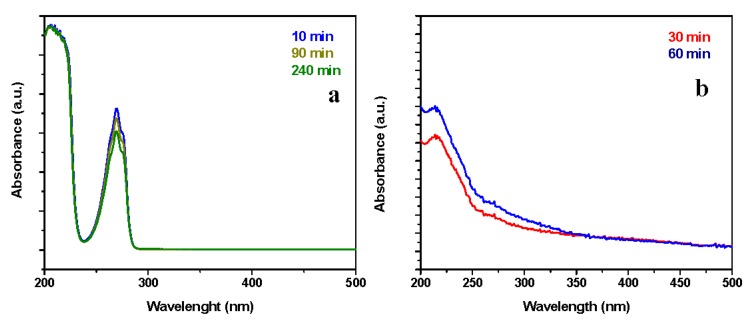
UV-spectra of the reaction media (reaction solutions at specific times) at 95 °C, 1000 mg/L phenol concentration, 0.4 g/L of HCL resin (**a**) and HCL nanocomposite (**b**).

A further test was performed at a lower phenol concentration, the neat resin was tested in the operating conditions: 95 °C, 0.3 MPa of air, airflow 4.6 NL/h, phenol concentration 1000 mg/L, resin concentration 0.4 g/L, Figure 11. The UV–vis spectra profiles, see Figure 12a, which are practically superimposable, except for the absorbance intensities, confirm the absence of catalytic phenomena for the neat resin. This result was further analyzed by GC-MS, which shows a single peak, at 19.95 min retention time, attributable to phenol. In this case, the result of the experiments is not directly comparable with that obtained at higher concentration, because we cannot exclude desorption (the catalytic resin has been saturated with 4000 mg/L of phenol solution, before test in the presence of 1000 mg/L of phenol). The UV–vis spectra of reaction mixtures at two different reaction times in the presence of the catalyst loaded resin are shown in Figure 12b. The absence of the typical phenol absorption bands suggests the depletion of phenol. In particular, the spectrum at 30 min already suggests the formation of low carboxylic acids (i.e., acetic acid) [21]. The results of the GC-MS, performed on the reaction solution, confirms the UV–vis evaluation (Figure 11), evidencing for example at 300 min of reaction a presence of non-reacted phenol (19.95 min retention time) which accounts for 3% of the total detected species, while acetic acid (retention time 14.17 min) was the other detected molecule, with a phenol removal of 99.9%. In these conditions, TOC conversion (ΔTOC) results equal to 97.1%, corresponding to a mineralization degree of about 96.8%.

The results are very relevant, also if compared with the literature [20,21,26,28,29,35,36,39,44,45], considering the pressure and temperature conditions and the low amount of noble metals used in our tests.

This is due to a combination of factors: (i) the adsorption ability of the resin towards organic chemicals (e.g., phenol), and probably oxygen, which results in a sort of reagents pre-concentration enabling reaction rate increase; (iv) the PtRu alloy activity; (v) the MoS_2_ nanosheets which are characterized by a high level of defects, that protects physically the catalyst surface against carbon polymerization and more, in general, chemically against poisoning (e.g., sulfur,..); and finally (vi) the defects in MoS_2_, including S vacancy (monosulfur and disulfur vacancies), and external Mo atom, which break the surface chemical inertness, enabling gases adsorption, e.g., oxygen reservoir [71].

Moreover, the active species was not supported on the resin surface but homogeneously dispersed into the polymeric matrix, preserving resin hierarchical porous construction (i.e., the combination of micropores and mesopores) and providing easy access channels for reaction medium. The NPs result confined in the hyper-crosslinked matrix, which preserves nano-dimensions, reduces leaching phenomena and the amount of active species required because of the total surface area exposed.

## 4. Conclusions

PtRu/MoS_2_ nanoparticles (NPs) were prepared through a scalable ‘wet chemistry’ approach and embedded, at 5 phr loading, in a poly (divinylbenzene-co-vinyl benzyl chloride) HCL resin.

TEM analysis of the NPs allows to evaluate their morphology, showing that NPs are, constituted of a PtRu core (mean size 4 nm–standard deviation 0.8 nm) partially covered by MoS_2_ nanosheets, mostly one-layer. In the sample MoS_2_ nanosheets alone can also be observed. Energy dispersive TEM based X-ray spectroscopy (EDS) confirms the Pt/Ru atomic ratio ~1.94. X-ray diffraction analysis evidences the successful formation of a PtRu alloy.

The HCL nanocomposite containing PtRu/MoS_2_ NPs shows good distribution of the nanoparticles. Moreover, in the nanocomposite, an increase of the micropores/mesopores ratio is observed concerning the neat HCL resin. Overall, phenol wet air oxidation tests evidence the quasi-complete successful removal of phenol and conversion in non-toxic products in the presence of the HCL nanocomposite containing PtRu/MoS_2_ NPs. The operating conditions (i.e., 95 °C; 0.3 MPa; and 0.4 g/L, 5 phr of the PtRu/MoS_2_ active species) indicate the excellent behavior of the nanocatalyst. This is particularly relevant also considering that the active species is not supported on the resin surface, but homogeneously dispersed into the polymeric matrix. Indeed, the embedding of the nanocatalyst preserves the hierarchical resin porosity (i.e., the combination of micropores and mesopores) leaving easy access to the channels for reaction medium. The syntheses result in confined NPs in the HCL resin, which reduces leaching phenomena and the amount of active species required because of total surface area exposed.

## Figures and Tables

**Table 1:** Operating conditions.

Parameters	Value
Temperature (°C)	95
Pressure (MPa)	0.3
Air Gas Flow (NL/h)	4.6
Phenol concentration (mg/L)	4000–1000
Catalyst concentration (g/L)	0.4

**Table 2:** GC-MS configuration.

GC-MS Configuration	
**Injector**	
Inlet temperature	230 °C
Sample size	1 µL
Split ratio	12
**Column temperature program**	
Initial temperature	50 °C for 1 min
Rate 1	7.0 °C/min to 180 °C for 1 min
Rate 2	10 °C/min to 300 °C for 1 min
**Detector**	
Type	Mass spectrometer
Interface temperature	230 °C
**Column**	
Type	HP-5 (0.25 µm × 0.25 mm × 30 m)
Flow rate	1 mL/min
**Other**	
Gas Flow	Helium
Electron ionization (EI)	70 eV
Scan range	35 ÷ 450 amu
Scan rate	1.80 scans/s

## Supplementary Materials

The following are available online at https://www.mdpi.com/2079-4991/9/10/1477/s1, Figure S1: FT-IR spectrum of free oleylammine.
