# Supplementary material for: A New Route for Low Pressure and Temperature CWAO: A PtRu/MoS2_Hyper-Crosslinked Nanocomposite
Source: Nanomaterials (Basel). 2019 Oct 17;9(10):1477. doi: 10.3390/nano9101477 (PMC6835422; doi:10.3390/nano9101477)
Supplement: Supplementary file 1 [file nanomaterials-09-01477-s001.pdf]

Supporting Information

A New Route for Low Pressure and Temperature CWAO: A PtRu/MoS_2_Hyper-Crosslinked Nanocomposite

Rachele Castaldo [1,†], Mariagrazia Iuliano [2,†], Mariacristina Cocca [1], Veronica Ambrogi [3], Gennaro Gentile [1], and Maria Sarno [2,*]

[1] Institute for Polymers Composites and Biomaterials, National Research Council of Italy, Via Campi Flegrei 34, 80078 Pozzuoli, Italy; rachele.castaldo@jpcb.cnr.it (R.C.); cocca@ictp.cnr.it (M.C.); gennaro.gentile@cnr.it (G.G.)

[2] Department of Industrial Engineering and Centre NANO_MATES University of Salerno, Via Giovanni Paolo II, 132-84084 Fisciano (SA), Italy; maiuliano@unisa.it

[3] Department of Chemical, Materials and Production Engineering, University of Naples Federico II, Piazzale Tecchio 80, 80125 Napoli, Italy; ambrogi@unina.it

† These authors contributed equally.to this paper.

* Correspondence: msarno@unisa.it; Tel.: +39-08-996-3460; Fax: +39-08-996-4057

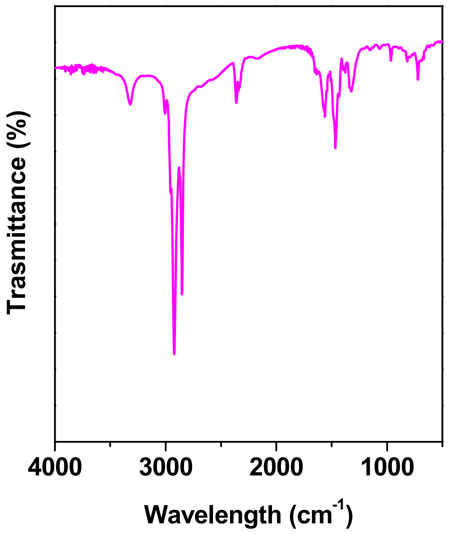

Figure S1. FT-IR spectrum of free oleylammine.
